# Dynamic Interactions of Post Cleaved NS2B Cofactor and NS3 Protease Identified by Integrative Structural Approaches

**DOI:** 10.3390/v14071440

**Published:** 2022-06-30

**Authors:** Jun-Ping Quek, Zheng Ser, Bing Liang Alvin Chew, Xin Li, Lili Wang, Radoslaw M. Sobota, Dahai Luo, Wint Wint Phoo

**Affiliations:** 1 Lee Kong Chian School of Medicine, Nanyang Technological University, EMB 03-07, 59 Nanyang Drive, Singapore 636921, Singapore; junping001@e.ntu.edu.sg (J.-P.Q.); alvin.chew@ntu.edu.sg (B.L.A.C.); 2 NTU Institute of Structural Biology, Nanyang Technological University, EMB 06-01, 59 Nanyang Drive, Singapore 636921, Singapore; 3 Functional Proteomics Laboratory, SingMass National Laboratory, Institute of Molecular and Cell Biology, Agency for Science, Technology and Research, 61 Biopolis Drive, #07-03, Singapore 138673, Singapore; ser_zheng@imcb.a-star.edu.sg (Z.S.); rmsobota@imcb.a-star.edu.sg (R.M.S.); 4 NTU Institute of Health Technologies, Interdisciplinary Graduate School, Nanyang Technological University, RTP 02-07, 50 Nanyang Drive, Singapore 637553, Singapore; 5 SingMass National Laboratory, Department of Biological Sciences, National University of Singapore, 14 Science Drive 4, Blk S2, #02, Singapore 117543, Singapore; xinli19@nus.edu.sg (X.L.); wll_ms@nus.edu.sg (L.W.); 6 School of Biological Sciences, Nanyang Technological University, 60 Nanyang Drive, Singapore 637551, Singapore

**Keywords:** NS2B-NS3 protease, DENV, dengue virus, dengue fever, viral protease, X-ray crystallography, cross-linking mass spectrometry

## Abstract

Diseases caused by flaviviruses such as dengue virus (DENV) and West Nile Virus (WNV), are a serious threat to public health. The flavivirus single-stranded RNA genome is translated into a polyprotein which is cleaved into three structural proteins and seven non-structural proteins by the viral and cellular proteases. Non-structural (NS) protein 3 is a multifunctional protein that has N-terminal protease and C-terminal helicase domains. The NS3 protease requires co-factor NS2B for enzymatic activity and folding. Due to its essential role in viral replication, NS2B-NS3 protease is an attractive target for antiviral drugs. Despite the availability of crystal structures, dynamic interactions of the N- and C-termini of NS2B co-factor have been elusive due to their flexible fold. In this study, we employ integrative structural approaches combined with biochemical assays to elucidate the dynamic interactions of the flexible DENV4 NS2B and NS3 N- and C-termini. We captured the crystal structure of self-cleaved DENV4 NS2B_47_NS3 protease in post cleavage state. The intermediate conformation adopted in the reported structure can be targeted by allosteric inhibitors. Comparison of our new findings from DENV4 against previously studied ZIKV NS2B-NS3 proteins reveals differences in NS2B-NS3 function between the two viruses. No inhibition of protease activity was observed for unlinked DENV NS2B-NS3 in presence of the cleavage site while ZIKV NS2B-NS3 cleavage inhibits protease activity. Another difference is that binding of the NS2B C-terminus to DENV4 eNS2B_47_NS3Pro active site is mediated via interactions with P4-P6 residues while for ZIKV, the binding of NS2B C-terminus to active site is mediated by P1-P3 residues. The mapping of NS2B N- and C-termini with NS3 indicates that these intermolecular interactions occur mainly on the beta-barrel 2 of the NS3 protease domain. Our integrative approach enables a comprehensive understanding of the folding and dynamic interactions of DENV NS3 protease and its cofactor NS2B.

## 1. Introduction

Flaviviruses are positive-sense single-stranded RNA viruses that comprise important human pathogens such as dengue virus (DENV), West Nile Virus (WNV), Japanese Encephalitis virus (JEV), and Zika virus (ZIKV). With 40% of the world population at risk, these pathogenic flaviviruses pose a serious threat to public health [1]. Flavivirus genome is 11 kb long with 3′ and 5′ untranslated regions [2]. The genome is translated into a polyprotein which is then cleaved by viral and cellular proteases into three structural proteins and seven non-structural proteins [2]. Non-structural protein 3 is a 69 kDa protein with N-terminal protease and C-terminal helicase domain [3]. The NS3 protease, a serine protease, requires NS2B as a co-factor for enzymatic activity and folding [3,4]. The NS2B-NS3 viral protease is responsible for polyprotein cleavages between NS2A/2B, NS2B/3, NS3/4A, and NS4B/5 [3,4,5]. Due to its essential role in the viral replication process, NS2B-NS3 protease is an attractive target for antiviral drugs.

Conventionally, the structural and biochemical studies of the NS2B-NS3 protease domain are mostly carried out using the engineered construct where the central cofactor region of NS2B is covalently linked to NS3 by a flexible glycine linker [6,7,8,9,10]. The crystal structures of the construct indicate that NS3 protease has a chymotrypsin-like fold with NS2B cofactor wrapped around NS3 similar to a belt [8,9]. The C-terminus of NS2B could either be dissociated from NS3 or fold back into β-hairpin and participate in catalysis [8,9]. The conformational exchange of NS2B between open and closed conformations has been observed by NMR studies as well [11,12,13]. The lack of well-dispersed NMR spectra of N2B-NS3 conventional construct has led to the utilisation of different construct designs to give heterodimer NS2B-NS3 proteases [14]. One of these construct designs is enzyme-linked construct where the cleavage NS2B/3 site was inserted between NS2B cofactor and NS3 [14,15]. The purified NS2B-NS3 undergoes self-cleavage resulting heterodimer of NS2B-NS3 protease. Another is the co-expression of NS2B cofactor and NS3 as two separate peptides [14,15]. The enzymatic activity of ZIKV NS2B-NS3 protease with three different linkers: (1) linker consisting of cleavage site between NS2B and NS3; (2) conventional flexible glycine linker (GGGGSGGGG); and (3) co-expression of NS2B and NS3 as separate peptides, have been compared [15]. The presence of either the NS2B/NS3 cleavage site or the flexible glycine linker has an inhibitory effect on the enzymatic activities of ZIKV NS3 protease [15]. On the contrary, biochemical assays performed in the DENV4 full-length NS3 studies with three different linkers similar to ZIKV b-, e- and gZiPro, indicates that presence of NS2B/3 cleavage site at the C-terminus of NS2B cofactor do not have an inhibitory effect on the protease activity nor inhibitor binding activity [16]. Despite the similarities in overall fold between protease domain of DENV4 NS3 full length and ZIKV eZiPro structures, the NS2B C-terminus in the crystal structure of DENV4 unlinked NS2B-NS3 full length is flexible in comparison to that of ZIKV eZiPro where the NS2B/3 cleavage pentapeptide occupy the active site [15,16]. In addition, the N- and C-termini of NS2B and NS3 regions are flexible in the crystal structures of NS2B-NS3 protease from various flaviviruses limiting the knowledge on the interaction dynamics of these regions. Integrative structural studies combining 3D molecular structures provided by X-ray crystallography with mapping of dynamic interactions via cross-linking mass spectrometry have been applied to provide a comprehensive understanding of the molecular folding and mechanisms of enzymatic action of the proteins [17,18,19].

In this study we combined biochemical assays with intact protein analysis by LC-MS, 3D molecular structure determination by X-ray crystallography and dynamic interactions mapping by cross-linking mass spectrometry (XL-MS) to map the interactions of post-cleaved NS2B-NS3 protease from DENV4. We designed NS2B-NS3 protease construct from DENV4 in similar manner to eZiPro. Six residues from NS2B/3 cleavage site were inserted between the NS2B cofactor and NS3 protease. We call the construct, eNS2B_47_NS3Pro. Combining LC-MS analysis and X-ray crystallography, we provide structural basis of NS2B C-terminus occupying NS2B-NS3 active site. We employ cross linking mass spectrometry to map the interactions of the flexible NS2B N- and C-termini and NS3 N-terminus. Our integrative structural studies provide new insights into folding and dynamic interactions of post cleaved DENV4 NS2B-NS3 protease as well as an integrative framework for a comprehensive structural analysis of NS2B-NS3 protease.

## 2. Results

### 2.1. DENV4 NS2B/3 Cleavage Site at NS2B C-Terminus Does Not Interfere with Protease Activity

**Figure 1:**
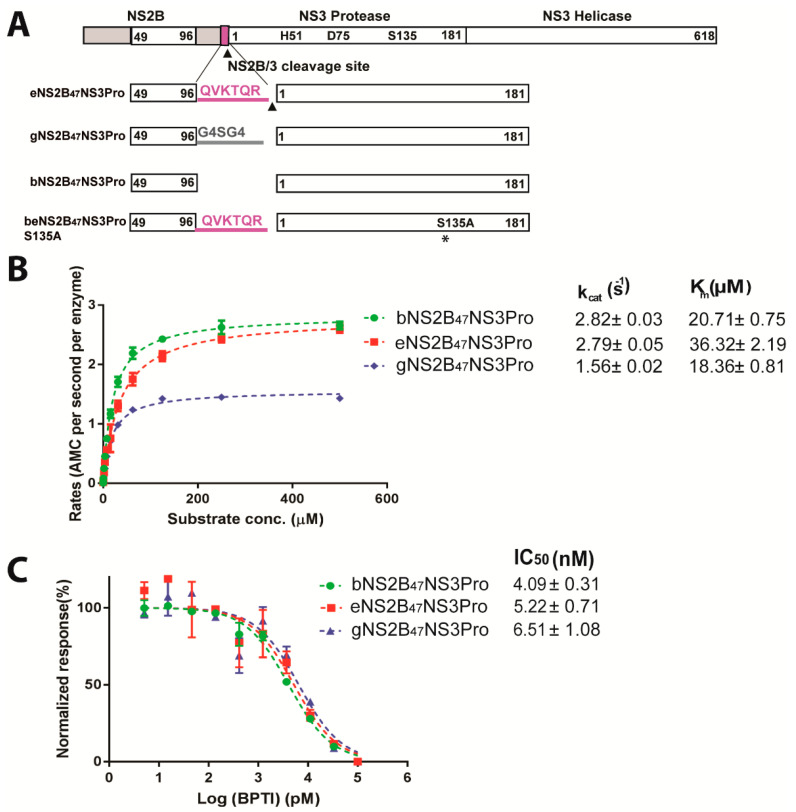
Schematic of construct design and characterization of protease activity from three different DENV4 NS2B_47_NS3 proteases. (**A**) Schematic of construct design of DENV4 NS2B_47_NS3Pro with three different linkers, e, g and b-. The beNS2B_47_NS3Pro S135A was created by inserting seven amino acids from NS2B/NS3 cleavage junction and by mutating the active serine residue 135 to alanine. The mutation, S135A, on beNS2B_47_NS3Pro is indicated in asterisk. (**B**) The protease activity of different NS2B_47_NS3Pro. The b- and e-NS2B_47_NS3Pro have similar *k_cat_* while gNS2B_47_NS3 Pro showed the lowest reaction rates. (**C**) The effect of different linkers on BPTI inhibition of the protease. The three constructs have similar inhibition efficiency indicating that the effect on linker on the substrate-binding is minimal in DENV. The standard deviations are represented by error bars.

To investigate the relationship between the DENV4 cleavage site and its protease activity, we designed NS2B-NS3 protease constructs from DENV4 (Figure 1A) in a similar manner to ZIKV protease constructs eZiPro, bZiPro and gZiPro from our previous study [15]. Construct eNS2B_47_NS3Pro had seven residues from the NS2B/3 cleavage site inserted between the NS2B cofactor and NS3 protease (Figure 1). Constructs gNS2B_47_NS3Pro and bNS2B_47_NS3Pro, with a flexible glycine linker and bivalent construct, respectively, were also generated (Supplementary Figure S1A). Construct beNS2B_47_NS3Pro S135A was made by inserting six residues from the NS2B/3 cleavage site at NS2B C-terminus and the serine residue 135 at the active site was mutated to alanine creating unlinked inactive protease with NS2B/3 cleavage site at NS2B C-terminus. SDS-PAGE analysis of the purified proteins from the different constructs showed that eNS2B_47_NS3Pro underwent complete proteolysis between the NS2B/NS3 junction resulting in a heterodimer of NS2B cofactor and NS3 protease similar to bNS2B_47_NS3Pro (Supplementary Figure S1A,B).

The protease activity of the DENV4 NS2B-NS3 protease constructs with different linkers was determined by a protease activity assay (Figure 1B). The protease activity of DENV4 eNS2B_47_NS3Pro and bNS2B_47_NS3Pro showed similar *k_cat_* values, with 2.82 ± 0.03 AMC molecule per second per enzyme for eNS2B_47_NS3 and 2.79 ± 0.05 AMC molecule per second per enzyme for bNS2B_47_NS3Pro, while gNS2B_47_NS3Pro had a slightly lower *k_cat_* with 1.56 ± 0.02 AMC molecule per second per enzyme (*p*-value < 0.01). The rate of catalysis (*k_cat_*) reported for ZIKV b-, e- and g- protease, bZiPro, eZiPro and gZiPro, are 5.302 ± 0.35, 1.79 ± 0.10, 1.073 ± 0.05 AMC molecule per second per enzyme [15]. Compared with ZIKV protease, the presence of NS2B/NS3 junction in DENV4 eNS2B_47_NS3Pro, bNS2B_47_NS3Pro and gNS2B_47_NS3Pro does not have a reduction in the rate of catalysis (*k_cat_*) (Figure 1B). The DENV4 eNS2B_47_NS3Pro has about two times higher *K_m_* (38.32 ± 2.19 µM) than that of b- (*K_m_* = 20.71 ± 0.75 µM) and g-NS2B_47_NS3Pro (*K_m_* = 18.36 ± 0.81 µM) probably due to the presence of NS2B/NS3 cleavage junction (*p*-value < 0.01).

Next, we used a protease inhibition assay to measure the binding efficieny of bovine pancreatic trypsin inhibitor (BPTI) to the DENV4 NS2B_47_NS3Pro enzymes. The IC_50_ values of BPTI for DENV4 bNS2B_47_NS3Pro, eNS2B_47_NS3Pro and gNS3B_47_NSPro are 4.09 ± 0.31 nM, 5.22 ± 0.71 nM and 6.51 ± 1.08 nM, respectively, whereas for ZIKV b-, e- and g-ZiPro, the IC_50_ values of BPTI were reported to be 12 nM, 350 nM and 76 nM, respectively, (Figure 1C) [15]. Hence, the presence of NS2B/NS3 cleavage site in eNS2B_47_NS3Pro and in eZiPro exerts different effects on the protease and inhibitor binding activities of NS2B/3 protease.

### 2.2. Molecular Weight Determination of DENV4 b-, e-, g-NS2B_47_NS3Pro Constructs by LC-MS

**Figure 2:**
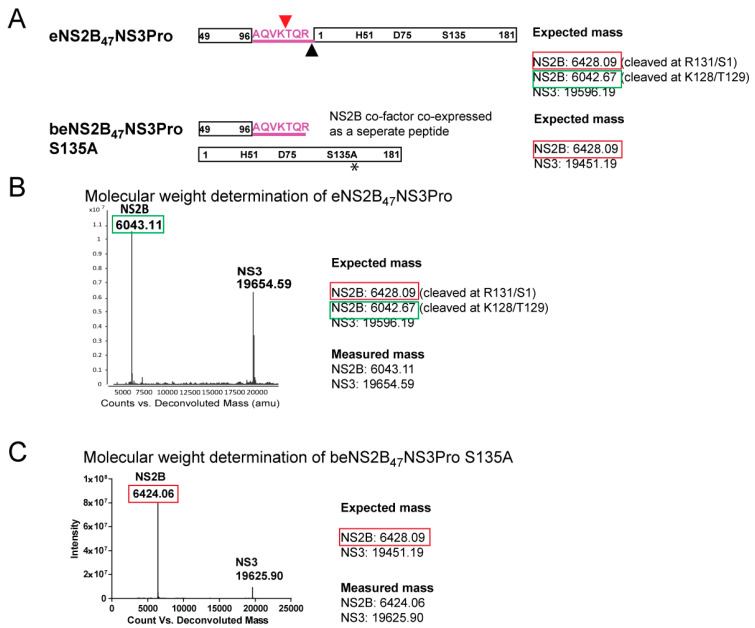
Molecular weight determination of active and inactive DENV4 NS2B_47_NS3 proteases. (**A**) The construct design of eNS2B_47_NS3Pro and beNS2B_47_NS3Pro S135A and the expected molecular weights of the peptides. The mutation, S135A, on beNS2B_47_NS3Pro is indicated in asterisk. (**B**) Deconvoluted mass spectra of eNS2B_47_NS3Pro. The mass of NS2B peptide is lower than the expected mass indicating the truncated form. (**C**) Deconvoluted mass spectra of beNS2B_47_NS3Pro S135A. The measured mass of NS2B peptide reflects the un-truncated form of NS2B in the absence of the protease activity.

Despite the presence of the NS2B-NS3 cleavge site, the biochemical activity of DENV4 eNS2B47NS3Pro is similar to that of bivalent construct, bNS2B47NS3Pro. To validate if the NS2B-NS3 cleavage site at the NS2B C-terminus from eNS2B47NS3Pro is intact, we determined the mass of DENV4 NS2B/3 protease with different linkers, eNS2B_47_NS3Pro-, beNS2B_47_NS3Pro S135A-, gNS2B_47_NS3Pro- and bNS2B_47_NS3Pro-by intact protein LC-MS. The expected masses of intact and truncated NS2B co-factor peptide from eNS2B_47_NS3Pro was depicted in Figure 2A. Further cleavage of NS2B C-terminus on eNS2B47NS3Pro would result in a smaller expected mass of the NS2B co-factor. The mass of NS2B co-factor in eNS2B_47_NS3Pro is determined to be 6042.48 Da instead of the expected mass of 6428.09 Da (Figure 2A,B). The reduced mass corresponds to further cleavage of NS2B C-terminus at **127VK/TQR131**. This second cleavage site has not been reported before and is possibly the reason that eNS2B_47_NS3Pro has similar enzymatic activities as unlinked bNS2B_47_NS3Pro. The detected mass of gNS2B_47_NS3 Pro corresponds to 25,796.42 Da indicating there is no unexpected cleavage (Supplementary Figure S3A). In the case of bNS2B_47_NS3Pro, a portion of NS3 protease was found to have a lower mass peptide (Supplementary Figure S3B). The truncation at K15/A16 at the N-terminus of NS3 protease will result in the reduce mass of 17,666.21 (Supplementary Figure S3B). It is possible that due to the lack of C-terminus NS2B/3 cleavage site, a portion of the NS3 has undergone further proteolysis over time. The molecular weight of ZIKV eZiPro NS2B C-terminus determined by intact protein LC-MS reflects the cleavage at **126VKTGKR131** with no further cleavage was detected [20].

To determine if the cleavage is due to unspecific bacterial proteases during protein expression in *E.coli*, construct beNS2B_47_NS3Pro S135A which co-expresses NS2B co-factor residues and NS3 protease was made (Figure 2A). Similar to eNS2B_47_NS3Pro, the beNS2B_47_NS3Pro S135A construct is made up of two peptides, NS2B co-factor and NS3 protease. Due to the S135A mutation, the beNS2B_47_NS3Pro S135A construct has no protease activity and therefore cis- processing of NS2B C-terminus is absent. The SDS PAGE analysis and gel filtration profile of purified protein showed that NS3 peptide from beNS2B_47_NS3Pro S135A migrated similarly as eNS2B_47_NS3Pro (Supplementary Figure S2A,C) and form heterodimers (Supplementary Figure S2B,D). The beNS2B_47_NS3Pro S135A construct was purified the same way as eNS2B_47_NS3Pro and the mass of the protein was identified in similar fashion. The molecular weight analysis reveals that the NS2B co-factor of bNS2B_47_NS3Pro S135A has a molecular weight of 6424.06 Da. The expected molecular weight of NS2B co-factor of bNS2B_47_NS3Pro S135A is 6428.09 indicating that the measured NS2B co-factor of bNS2B_47_NS3Pro S135A is intact and second cleavage is not detected suggesting that the NS2B-NS3 protease is responsible for the truncation of NS2B C-terminus (Figure 2A,C).

**Figure 3:**
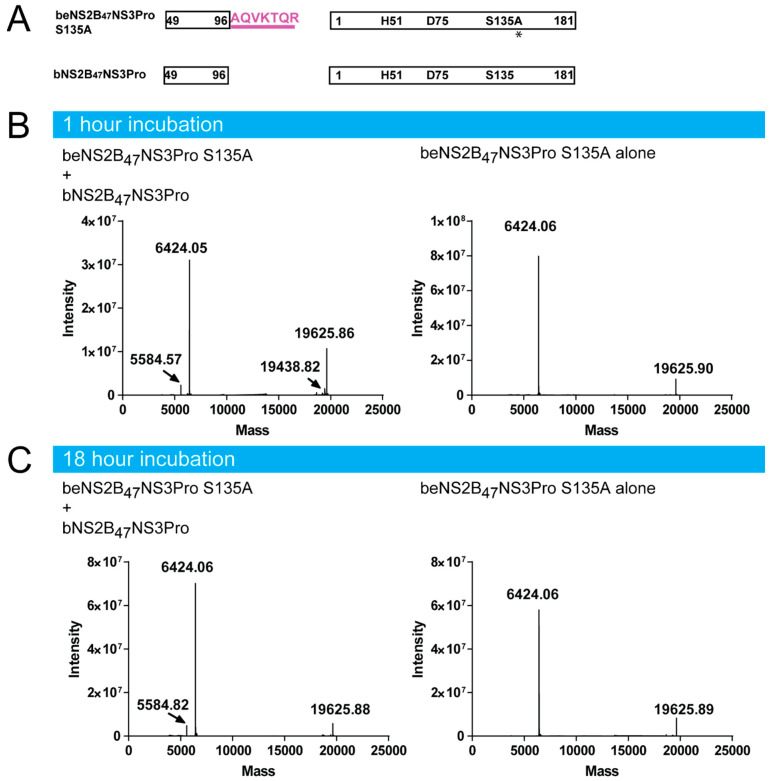
The cleavage between DENV4 NS2B and NS3 occurs in cis. (**A**) Schematic diagram active and unlinked DENV NS2B_47_NS3 Pro constructs. The mutation, S135A, on beNS2B_47_NS3Pro is indicated in asterisk. (**B**) The molecular weight determination of beNS2B_47_NS3Pro S135A with and without active bNS2B_47_NS3 Pro at two-time points (**B**) after 1 h incubation (**C**) after 18 h incubation. The NS2B C-terminus of beNS2B47NS3Pro S135A stays intact even after 18 h of incubation with active unlinked NS3 protease, bNS2B_47_NS3 Pro indicating that the cleavage of NS2B C-terminus is mediated in cis.

To determine if the second cleavage of NS2B terminus happens in trans, we incubated the beNS2B_47_NSPro S135A with active protease bNS2B_47_NS3Pro in 10:1 ratio at two time points, 1 h and 18 h (Figure 3A–C). The masses of the proteins were subsequently measured by intact protein LC-MS. The analysis showed that no cleavage of NS2B from beNS2B_47_NS3Pro S135A was detected, while we could detect the truncated NS2B from bNS2B_47_NS3Pro in lower intensity (Figure 3B,C). These results confirm that the second cleavage of NS2B C-terminus is mediated exclusively via cis-cleavage of NS2B-NS3 protease.

**Figure 4:**
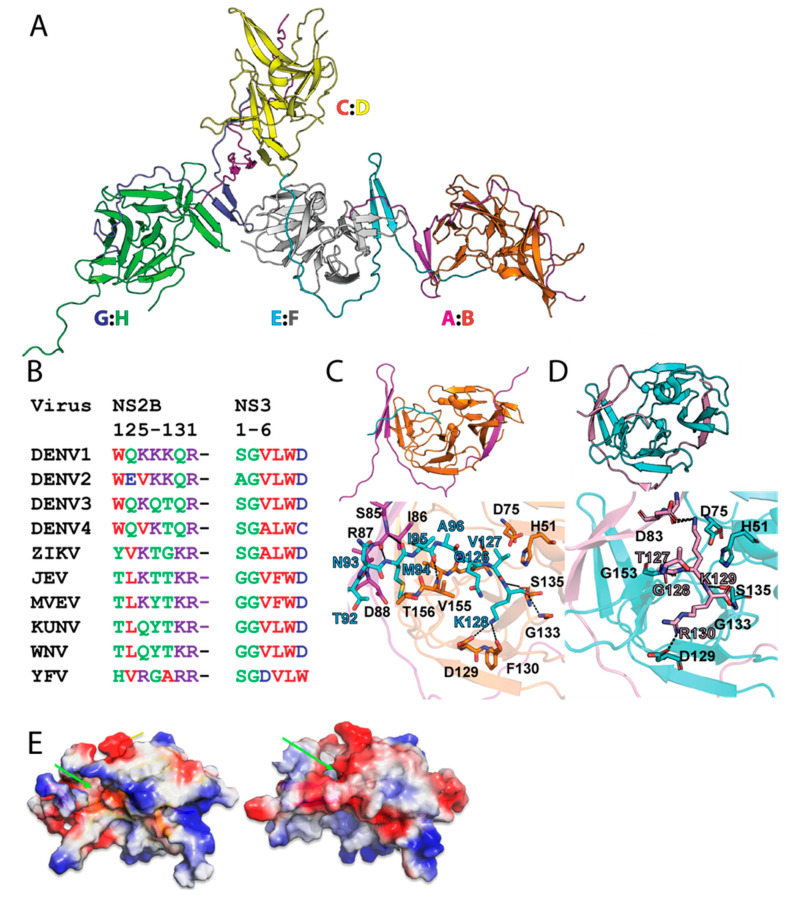
Crystal structure of eNS2B_47_NS3Pro reveals a novel conformation of NS2B cofactor. (**A**) One unit cell of eNS2B_47_NS3Pro shows the two dimer units. The NS3 domains are annotated as (B,D,F,H) and NS2B cofactor as (A,C,E,G). The chains’ IDs are indicated next to the molecule. (**B**) The sequence alignment of the NS2B-NS3 cleavage site for different flaviviruses. (**C**,**D**) Comparison between the active site of NS2B-NS3 protease from DENV4 (**B**) and ZIKV (**C**). The residues involved in the interactions are labelled and shown as sticks. The interacting residues are numbered and labelled with one amino acid code. The partially closed conformation of DENV NS2B-NS3 protease creates a larger active site allowing P4-P6 substrate residues to form interactions with NS2B. (**E**) Surface charge representation of DENV protease. Left: Partially closed conformation in eNS2B_47_NS3Pro. A small hydrophobic binding site could be observed during the partial opening of NS2B cofactor. Right: Fully closed conformation of NS2B cofactor (PDB: 3U1I).

Crystal structure of eNS2B_47_NS3Pro shows a partially closed NS2B conformation. To provide the structural basis of the self-cleaved eNS2B_47_NS3Pro, the crystal structure of eNS2B_47_NS3Pro was determined at 3.35 Å resolution. The data collection statistics are shown in Table 1 and the structure has been deposited with PDB code 7VMV. The structure showed two dimers in one unit cell (Figure 4A). The overall fold of individual NS3 protease molecule is similar with root mean square deviation (RMSD) less than 0.3 Å (Supplementary Figure S4). Superimposition of eNS2B_47_NS3Pro structure on previously reported DENV protease structures showed similar overall fold of NS3 protease (Supplementary Figure S5A).

In our structure, the NS2B cofactor adopts a new conformation, where the NS2B C-terminus is not fully folded back as β-hairpin (Figure 4A, Supplementary Figure S4A). Instead, the NS2B C-terminus occupied the active site of neighbour NS3 protease in an adjacent unit cell due to the presence of the C-terminal NS2B/NS3 cleavage peptide sequence (Figure 4A). This is in contrast with the previously reported crystal structures of NS2B-NS3 protease where they exist as either closed conformation or open conformation [8,9,15,20]. In the current structure, the cleaved C-terminus of NS2B (**126QVK128**) enter the neighbouring protease unit via a side tunnel and occupy the active site of the NS3Pro of the dimer unit (Figure 4A–C). The C-terminal carboxyl oxygen forms close contact with the side chain of S135 and amide nitrogen of G133 (Figure 4C). The P_1_ amide NH also forms hydrogen bonding with the hydroxyl oxygen of S135 and the carbonyl oxygen of G151. The nitrogen on the side chain amine of P_1_ lysine interacts with the side-chain oxygen of D129 and the carbonyl oxygen of F130 (Figure 4C). The P_3_ glutamine carbonyl oxygen and amide NH interact with the G153 amide NH and carbonyl oxygen, respectively. The P_5_ isoleucine carbonyl oxygen and amide NH interact with the V155 amide NH and carbonyl oxygen, respectively. In the case of self cleaved ZIKV protease structure, eZiPro, the NS2B C-terminus although found to be intact by intact protein LC-MS analysis, is flexible and only 4 residues occupying the active site were observed.

In both eNS2B_47_NS3Pro and eZiPro, NS2B C-terminus occupy the active site of the protease (Figure 4C,D). Yet, the interactions between the NS2B C-terminus and NS3 active site differ between the two structures In eNS2B_47_NS3Pro, P1 residue, K128 interacts with the active site residues D129, F130 and G133 (Figure 4C). This constrasts with eZiPro where both side chains and backbones moieties from the P1–P3 residues, **129GKR131**, interact extensively with both NS2B and NS3 residues lining the active site [15]. The side chains of P2 and P3 residues, V127 and Q126, are pointing away from the substrate site and do not interact with the substrate binding site while the P4–P6 residues, **94MIA96** form interactions with nearby residues from NS2B and NS3 (Figure 4C).

The NS2B/NS3 cleavage site of all four serotypes of dengue contain conserved glutamic acid residue at the P2 position while NS2B/NS3 cleavage site for other flaviviruses such as ZIKV, YFV, WNV and MVEV, contains conserved and strongly basic lysine or arginine as P2 residue (Figure 4B). The conservation of non-optimal glutamic acid as P2 residues suggest that DENV may regulate the NS2B-NS3 protease activity via weak suboptimal substrate binding. The most variable regions of the protease are NS3 loop 111–126 and loop 151–162. These two loops seem to act as hinges during the cofactor movement from open to close conformation (Supplementary Figure S5B).

When we compare the surface charge density of eNS2B_47_NS3Pro with that the published structure of DENV2 gNS2B_47_NS3 Pro in the closed conformation (PDB ID: 3U1I), we observe a small hydrophobic pocket between the NS2B and NS3 interface in our partial closed conformation comprising of residue I161–A164, residues R74–L76, residues G148–N152 (Figure 4E). Maus et al., has reported novel benzothiazoles as allosteric inhibitors of DENV NS2B-NS3 protease [21]. The predicted binding mode of these inhibitors indicates the same hydrophobic pocket that is reported in the current structure. As there is a lack of protein constructs and crystal structures available for capturing the protease with allosteric inhibitors, this construct of NS3 protease with the partially associated NS2B is useful for future co-crystallization/screening experiments with allosteric inhibitors especially to confirm the putative binding site of these allosteric inhibitors To gain detailed molecular interactions between the allostersric inhibitors and the protease, the resolution of the 3D molecular structure should be further improved.

### 2.3. Mapping the Long-Range Interactions between DENV4 NS2B and NS3 Protease via Cross-Linking Mass Spectrometry

**Figure 5:**
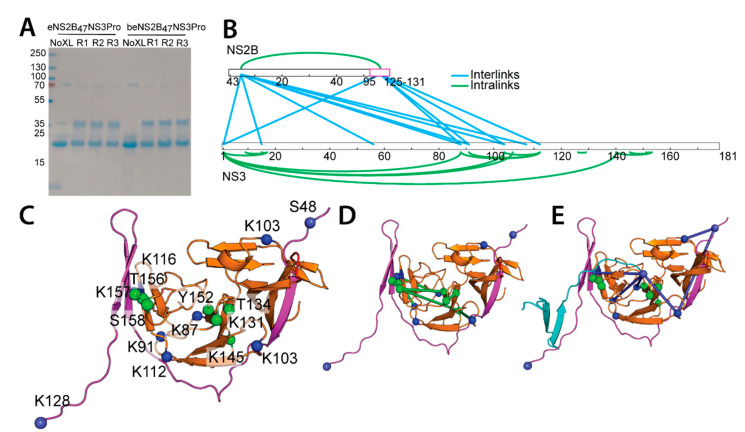
Mapping of dynamic interactions between DENV4 NS2B and NS3 via crosslinking mass spectrometry. (**A**) SDS PAGE analysis of eNS2B_47_NS3Pro and beNS2B_47_NS3Pro S135A before and after crosslinking with DSSO. The proteins in cross-linked samples migrated to higher band in SDS PAGE reflecting their higher molecular weight. (**B**) Residues from NS2B and NS3 that are mapped as intra and inter molecular crosslinks. (**C**) The identified crosslinks residues are mapped to eNS2B_47_NS3Pro structure (PDB id 7VMV). NS2B is shown in magenta, NS3 protease in orange, residues that are cross-linked with NS2B N- and C-termini are in blue. The intra-molecular cross-linked NS3 residues are shown in green spheres. The residue number for NS2B and NS3 cross-linked residues are described. The hydrophobic loop of NS3 (^29^GLFG^32^) is shown in red. (**D**) The NS3 intra-molecular cross-links are depicted in green lines. (**E**) The inter-molecular cross-links between NS2B and NS3 are shown in blue lines. The eNS2B_47_NS3Pro dimer is used to model the inter-molecular cross-links. The NS2B from the neighbouring molecule (chain ID: **E**) is depicted in cyan, the NS2B is in magenta and NS3 protease is in orange.

To understand the interactions of the disordered regions of the N- and C-terminal residues of DENV4 NS2B and N-terminal residues of NS3, we utilized in solution cross-linking mass spectrometry to map the proximal interacting regions of NS2B and NS3 proteins. Cross-linking of NS2B-NS3 complex was performed using an amine-reactive MS-cleavable disuccinimidyl sulfoxide (DSSO), with a spacer arm length of 10.3Å, and confirmed by SDS PAGE analysis with an observed protein band corresponding to the sum of the molecular weights of NS2B and NS3 (Figure 5A).

In total, 32 and 33 unique cross-links were identified for eNS2B_47_NS3Pro and beNS2B_47_NS3Pro S135A, respectively, across 3 replicates, with 60–70% of cross-links identified in 2 or more replicates (Supplementary Figure S6A,B, Figure 5B, Supplementary File S1). Between eNS2B_47_NS3Pro and beNS2B_47_NS3Pro S135A, 22 common cross-links are found. This indicates that the enzymatic activity has little influence on the intra- and inter-molecular interactions of NS2B-NS3 protease. The identified cross-links were mapped to the eNS2B_47_NS3Pro structure with 70% of cross-links within 30Å Cα-Cα distance, indicating a good agreement between the in-solution cross-linking structure and the crystal structure. The cross-links confirm the overall structure of the NS2B-NS3 complex, with the NS2B N-terminus cross-linked to the NS2B C-terminus.

Among the cross-link pairs identified, 9 pairs fell on a flexible region of NS3 N-terminus whose electron density was not visible in the crystal structure (Figure 5B). These NS3-NS3 cross-links are between residues 90–120 from the second β-barrel of chymotrypsin-like fold of NS3 protease (Figure 5B–D). Cross-links between NS2B and NS3 protease revolve around S48 on the N-terminus and K128 at the C-terminus of NS2B (Figure 5C,E). The NS2B-NS3 cross-links NS2B interacts with NS3 protease at (1) at the N-terminus of NS3 (2) at amino acids K87, K91 of NS3 (3) near or at the loop region of residue 110–120 of NS3 (Figure 5B,C).

**Figure 6:**
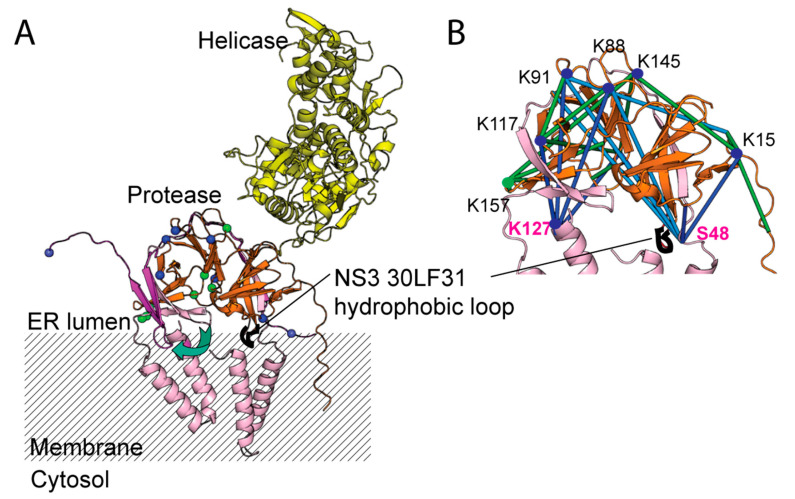
Model of full length DENV4 NS2B and NS3 on ER membrane. (**A**) eNS2B_47_NS3Pro structure is superimposed on full length DENV4 NS2B-NS3 predicted by AlphaFold. The NS3 helicase is coloured in yellow and protease in orange, the predicted NS2B full length is in light pink while the NS2B cofactor from eNS2B_47_NS3Pro structure is in magenta. The residues involved in intra-molecular cross-links are in green and the inter-molecular cross-linked residues are in blue. The hydrophobic loop of NS3 (^29^GLFG^32^) is shown in red. (**B**) The NS2B-NS3 protease is zoomed and the inter- and intra-molecular crosslinks are modelled on the NS2B-NS3 protease domain from AlphaFold model. The inter-molecular cross-links are in green and intra-molecular cross-links are in dark blue. The inter-molecular cross-links with Cα-Cα distance larger than 30Å are depicted as light blue.

To map the interaction of post cleaved NS2B-NS3 protease with full length NS2B-NS3, we predicted the 3D model of full length NS2B-NS3 full length using AlphaFold2. The identified cross-links can be mapped to the predicted model with more than 80% cross-links within 30Å ± 3Å Cα-Cα distance (Figure 6). Similar to the crystal structure, the intra-NS3-NS3 cross-links are located away from the ER membrane exposed side of NS3 protease (Figure 6A,B). Previous studies have mentioned that NS3 protease is anchored to the membrane by a loop with two hydrophobic leucine and phenylalanine at residues 30 and 31 [16,22] (Figure 6A,B). Interestingly, the hydrophobic hairpin loop comprising residues L3031F is located on the β-barrel 1 with fewer cross-linking interactions (Figure 6A,B).

## 3. Discussion

Although many structural insights are available from crystal structures of different flavivirus protease, the N- and C-termini of NS2B cofactor and NS3 N-terminus are flexible limiting the knowledge on the interaction dynamics of these regions. In our previous studies, ZIKV protease constructs with different linkers between NS2B-NS3 have varying biochemical activities [15]. The ZIKV construct with the artificial glycine linker and the one with the linker comprising 5 amino acid cleavage site between ZIKV NS2B-NS3 (NS2B residue number ^127^KTGKR^131^) have much higher *K_m_* compared to the bi-valent construct (bZiPro). This trend was not observed in DENV4 NS2B-NS3 full-length constructs with similar linkers, b-, e-, and g- [16] indicating that NS2B-NS3 cleavage site has differential effect on the enzymatic activity of NS2B-NS3 protease between DENV and ZIKV.

In this study, we designed NS2B-NS3 protease construct from DENV4 in a similar manner to eZiPro, bZiPro, and gZiPro. Seven residues from the NS2B/3 cleavage site were inserted between the NS2B cofactor and NS3 protease. We call the construct, eNS2B_47_NS3Pro. A construct with a flexible glycine linker and bivalent construct were also created namely, gNS2B_47_NS3Pro and bNS2B_47_NS3Pro. The six residues from the NS2B/3 cleavage site were inserted at NS2B C-terminus and the serine residue 135 at the active site was mutated to alanine creating unlinked inactive protease with NS2B/3 cleavage site at NS2B C-terminus. The construct was named beNS2B_47_NS3Pro S135A. Agreeing with a previous study on DENV4 NS3 full length, protease activity of NS3 protease was not severely affected by the presence of NS2B/3 cleavage junction in contrast to the NS2B-NS3 protease from ZIKV [16]. Using molecular weight analysis of intact proteins using LC-MS, we identified the presence of a second cleavage site at 3 amino acid upstream (K128/T129) of the conventional NS2B/NS3 cleavage site (R131 NS2B/S1 NS3) in eNS2B_47_NS3Pro. We speculate that it is the second cleavage since the NS3 peptide of eNS2B_47_NS3Pro does not have extra amino acids from NS2B/NS3 cleavage junction. Hence, the cleavage between R131 from NS2B and S1 from NS3 may have happened prior to the cleavage between K128/T129. The cleavage of NS2B/3 junction by NS2B-NS3 protease has been shown to occur exclusively in cis- for both DENV and WNV [23,24]. The NS2B C-terminus of inactive beNS2B_47_NS3Pro does not undergo proteolysis after incubation with active protease bNS2B_47_NS3 Pro indicating the cleavage between K128 and T129 is mediated via cis-proteolysis.

The crystal structure of eNS2B_47_NS3Pro with K128 of NS2B occupying at the protease active site of neighbouring NS2B-NS3 molecule further confirms the cleavage at K128 (Figure 4A,C). The structure of DENV4 NS2B-NS3 protease after self-cleavage was reported with NS2B cofactor in a partially closed conformation (Figure 4A). The similar conformation of NS2B-NS3 has been predicted as one of the major conformations of NS2B-NS3 using MD simulations [25]. The partially closed conformation of NS2B cofactor in eNS2B_47_NS3Pro enable the C-terminal end of NS2B to extend further into the active site of neighbouring NS3 protease. In our previous studies, the NS2B C-terminus tetrapeptide from self-cleaved ZIKV NS2B NS3 protease resides in the active site of the same molecule. In the eNS2B_47_NSPro structure, the IAQVK peptide of NS2B C-terminus is found in the active site of neighbouring molecule, indicating that NS2B/3 cleavage site in DENV could dissociate from active site, unlike ZIKV eZiPro (Figure 4B–D). This could be why the enzymatic activity of eNS2B_47_NSPro is similar to bNS2B_47_NS3Pro. The occupation of the IAQVK peptide from DENV NS2B C-terminus is mediated by interactions between the P4-P6 residues to NS2B **85SIRD88** and NS3 **155VT156** residues and P1 K128 to NS3 **129DF130** (Figure 4B,C). In contrast, the TGKR peptide from ZIKV NS2B C-terminus occupy the active site via interactions between P1–P3 residues, GKR, to NS3 D129, G133 and S135 (Figure 4B,D). As a suboptimal substrate, glutamic acid and valine side chains of P2 and P3 from eNS2B_47_NS3Pro are rotated away from the active site. Therefore, the eNS2B_47_NS3Pro structure indicates that binding of suboptimal peptides is mediated via P4-P6 residues (Figure 4B–D). We also identified a small hydrophobic pocket that is exposed only when the NS2B is in the partial closed conformation (Figure 4E). Allosteric inhibitors potentially targeting the pocket have been reported [21]. Hence, the structure reported here is suitable for co-crystallization/docking with allosteric inhibitors.

To further shed light on the flexible DENV4 NS2B N- and C- termini, we employed cross-linking mass spectrometry to map the regions of DENV4 NS3 protease that are interacting with NS2B N- and C-termini. All of the cross-links on NS2B are exclusively located at the N- and C-termini of NS2B (Figure 5B). The outer beta strands of NS3 protease (residues 90–120) are consistently involved in the interactions with both NS2B N- and C-termini as well as with NS3 N-terminus (Figure 5B,C). The majority of the interactions between NS2B N- and C-termini with NS3 are specifically located in β-barrel 2 from the chymotrypsin-like fold of NS3 protease (Figure 5B,C,E). Interestingly, the hydrophobic hairpin loop comprising residues **30**LF**31** is located on the β-barrel with no crosslink interactions (Figure 6A) [22]. These findings suggest the dynamic interactions between NS2B N- and C-termini with NS3 protease are limited to the solvent-accessible part of the NS3 protease where the β barrel 2 is located (Figure 6). The NS3 N-terminus is interacting with residues K87, K103, K106 and K145 located on the back of the active site (Figure 6). Therefore, upon processing NS2B/NS3 cleavage site, NS3 N-terminus may wrap around the NS3 protease from behind the active site (Figure 6). Hence, our cross-linking analysis indicates that both intra- and inter-molecular interactions of DENV4 NS2B-NS3 protease is limited mainly to the β barrel 2 of NS3 protease.

In conclusion, integrative structural approaches combined with biochemical assays were utilized to elucidate the interactions of NS2B-NS3 protease from DENV4. We identified a novel cis-cleavage site between NS2B/NS3. Mapping of NS2B N- and C-termini with NS3 indicates that these intermolecular interactions occur mostly on the solvent-exposed β-barrel of the NS3 protease domain. Intramolecular interactions between NS3 protease and its N-terminus suggest that following the cleavage between NS2B/3, the N-terminus wraps around the NS3 protease from behind the active site. We report the crystal structure of DENV4 unlinked NS2B-NS3 protease at post processed stage. The structure can be applied for the screening of allosteric inhibitors. The biochemical and structural studies of DENV NS2B-NS3 protease and those of ZIKV NS2B-NS3 protease suggest that following the polyprotein processing at NS2B/3 cleavage site by NS2B-NS3 protease, DENV NS2B C-terminus may regulate the NS2B-NS3 protease activity via weak trans-inhibition while ZIKV NS2B C-terminus may do so via stronger cis-inhibition. Our integrative approach provides new insights into the folding and dynamic interactions of DENV NS3 protease and its cofactor NS2B post cleavage state.

## 4. Materials and Method

### 4.1. Protein Purification

All of the protein expression plasmids were transformed into Escherichia coli Rosetta T1R. An overnight starter culture was prepared by growing the transformants in LB medium, supplemented with appropriate antibiotics, at 37 °C for 18 h. The overnight culture will be used to grow LB medium until OD_600_ of 0.8–1.0 was reached. Subsequently, 1 mM Isopropyl β-D-1-thiogalactopyranoside (IPTG) was added to induce protein overexpression for 18 h at 18 °C. Cells were harvested via centrifugation at 6000 rpm for 10 min at 4 °C.

Cells were resuspended in resuspension buffer (1x phosphate-buffered saline (PBS), supplemented with 160 mM Sodium chloride (NaCl), 5% glycerol and 5 mM β-Mercaptoethanol (β-ME)). The cells were lysed by sonication at 70% intensity using a 5-sec on/off cycle for 5 min. Subsequently, the cells were subjected to another round of lysis via the homogenizer (GEA) at 900–1000 bar for 15 min. To remove the unbroken cells and cell debris, centrifugation of the lysate was carried out at 35,000 rpm for 1 h at 4 °C. The supernatant was collected. Ni NTA Beads 6FF beads (Bio Basic Asia Pacific Pte Ltd., Singapore) equilibrated with resuspension buffer was incubated with the supernatant for 1.5 h at 4 °C with slight agitation. The beads were then washed with 10 column volume (CV) of resuspension buffer supplemented with 20–40 mM imidazole. Finally, the protein of interest was eluted with 5 CV of resuspension buffer with 300 mM imidazole. The N-terminal Histidine affinity tag was cleaved by TEV protease and dialyzed overnight in the 1x PBS dialysis buffer as per manufacturer protocol. The cleaved His-tag was removed by flowing the dialysed through Ni-NTA agarose beads and the flow-through fraction was collected. The fractions were concentrated and purified using HiLoad 16/600 Superdex 75 pg (GE Healthcare, Chicago, USA) column with SEC150 buffer (25 mM HEPES, pH 7.5, 150 mM NaCl, 2 mM DTT, 5% glycerol). Fractions were pooled, concentrated using Amicon^®^ Centrifugal Filter Units with 10 kDa molecular weight cut-off via centrifugation at 3500× *g*. The purified proteins were aliquoted and flash-frozen with liquid nitrogen for storage.

### 4.2. Protease Activity Assay

The protease assays were carried out using peptide substrate with a flurophore, Bz-nKRR-AMC (Peptide Institut, Osaka, Japan). The protease cleaves the substrate releasing the fluorophore, AMC. The AMC could be detected by excitation at 380 nm and emission at 450 nm. The assay buffer used is 20 mM Tris HCL pH 8.5, 2 mM DTT, 10% glycerol and 0.01% Triton X 100. The buffer is filtered with Millipore filter with 0.2 µm pore size. The purified proteins are diluted to 4.5 nM in the assay buffer and 20 µL of diluted protein is added to the Corning^®^ 96 well half area black plates (Corning, Corning, NY, USA). The final concentration of protein in the assay was 3 nM. The different substrate concentrations ranging from 0–300 µM were added to the enzyme and the fluorescent readings are measured at 37 °C at one-minute interval over five minutes using Cytation 3 Multimode plate reader (BioTek, Winooski, VT, USA). The assays were carried out as triplicates. The AMC standard curve is determined by the relative fluorescent units against different molar concentrations of AMC. The relative fluorescent units (RFU) are converted to the molar amount of AMC released according to the AMC standard curve. Initial velocities were calculated using the following equation. Rate constant (*k_cat_)* was calculated from *V_max_* divided by enzyme concentration [E_t_]. Data were analysed and plotted using the Michalis-Menten equation with GraphPad Prism version 7.00 for Windows, GraphPad Software, San Diego, CA, USA.

### 4.3. Protease Inhibition Assay

The inhibition assay was carried out using the same substrate, Bz-nKRR-AMC. The BPTI in different concentration ranges were incubated with 3 nM protease DENV protease) for 30 min at room temperature. The reaction was initiated by the addition of AMC substrate at 20 µM. The initial velocities in RFU are recorded at 30 s intervals for 5 min. The protease with no inhibitor was used as a positive control, while the protease alone with no inhibitor and substrate was used as a negative control. The final assay volumes were 30 microliters. To plot the inhibition efficiency curve, the initial velocities were normalized with positive control as 100% and negative control as 0% using Normalise function in GraphPad. The inhibitor concentrations were transformed into a logarithmic scale using the Transform function in GraphPad Prism. Finally, the log [inhibitor]/% of inhibition function was used to determine the IC_50_s of the inhibitors using GraphPad Prism.

### 4.4. Crystallization

The eNS2B_47_NS3Pro crystals, with a final protein concentration of 40 mg/mL, were grown at 20 °C with a reservoir solution (1.7 M Sodium malonate, pH 5.0) with the hanging drop vapour diffusion set-up. The crystals were dehydrated and were cryoprotected using the reservoir solution supplemented with 20% glycerol. The crystals were flash-freeze and stored in liquid nitrogen and shipped to MXII beamline at Australian Light Source for data collection. Diffraction intensities were integrated using iMOSFLM 9. Scaling and merging of the intensities were carried out using software POINTLESS and AIMLESS from CCP4 suite [26]. Molecular replacement was performed using the program PHASER MR, with the protease domain of DENV4 NS2B18NS3 full length (PDB ID: 2VBC) as the search model. The model was subjected to multiple rounds of manual rebuilding using COOT and refinement using the PHENIX REFINE [27,28,29]. Data collection and refinement statistics are summarized in Table 1. The structure was deposited in the Protein Data Bank with PDB ID (7VMV).

### 4.5. eNS2B_47_NS3Pro S135A Mutagenesis

The eNS2B_47_NS3Pro S135A mutation was generated using the NEB Q5 site-directed mutagenesis kit (New England Biolabs, Ipswich, MA, USA) according to the manufacturer’s protocol. The transformed colonies were sub-cultured overnight in Luria Broth (LB) with appropriate antibiotics at 37 °C. The plasmids were purified using AxyPrep Plasmid Miniprep Kit (Axygen, Corning, NY, USA) and sent for verification and Sanger sequencing (Bio Basic Asia Pacific Pte Ltd., Singapore). All of the primers used in the experiments are summarized in Table 2.

### 4.6. beNS2B_47_NS3Pro Mutagenesis

The bivalent eNS2B_47_NS3Pro was generated in the lab by adding a fragment with a stop codon, T7 promoter and IRES with another start codon. Firstly, the linear vector backbone was generated via PCR using CloneAmp™ DNA polymerase (Takara, Shiga, Japan). Subsequently, the linear backbone was purified using the Monarch^®^ PCR & DNA Cleanup Kit (New England Biolabs, Ipswich, MA, USA) according to the manufacturer’s protocol. Next, the fragment insertion was performed using In-Fusion^®^ HD Cloning Kit (Takara, Shiga, Japan) according to the manufacturer’s protocol. The transformed colonies were sub-cultured overnight in LB with appropriate antibiotics at 37 °C. Then, the plasmids were purified from transformed bacterial colonies using AxyPrep Plasmid Miniprep Kit (Corning, Corning, NY, USA) and sent for verification and Sanger sequencing (Bio Basic Asia Pacific Pte Ltd., Singapore). All of the primers used in the experiments are summarised in Table 2.

### 4.7. beNS2B_47_NS3Pro S135a Digestion by bNS2B_47_NS3Pro

100 μg of beNS2B_47_NS3Pro S135A was incubated with 10 μg of bNS2B_47_NS3Pro for 1 h and 18 h in assay buffer (20 mM Tris pH 8.5, 10% glycerol, 0.01% Triton X-100). Next, the samples were subjected to acetone precipitation using 4 volumes of ice-cold acetone and spun down at 10,000 g for 20 min twice.

### 4.8. Intact Protein LC-MS Analysis and Data Processing

The analysis was done on a Q Exactive Plus Biopharma system coupled with Vanquish UHPLC (Thermo Fisher Scientific, Bremen, Germany). 2 µL of each sample was injected and separated on a 10 cm Thermo Scientific MAbPac column using 8 min gradient. The following experimental parameters were used on the Orbitrap platform: the instrument was calibrated externally using Thermo Scientific Pierce Calibration Solutions. The source was operated in positive mode, the capillary voltage was set to 3.8 kV, the capillary temperature was 325 °C. MS spectra were recorded at a resolution of 140,000 with a mass range 450–2500 m/z. The AGC is set to 3 × 10^6^ with 250 ms maximum injection time. The spectra were analysed by the BioPhama Finder 3.2 software using the Xtract algorithm for isotopically resolved spectrum (Thermo Fisher Scientific, Waltham, MA, USA). The deconvolution settings are: Fit Factor: 70%, Remainder Threshold: 25%. Since some of the peaks are low abundant, the minimum detected charge is set at 1 (numbers of charge states).

### 4.9. Cross-Linking Mass Spectrometry and Data Analysis

The cross-linking protocol was adapted from previously published protocols. [30,31] Purified eNS2B_47_NS3Pro and beNS2B_47_NS3Pro (45 µg) proteins were cross-linked in triplicates in 50 mM HEPES, pH7,4, 150 mM NaCl, 5 mM TCEP, 5% glycerol buffer with 1 mM DSSO (26:1 ratio for DSSO:protein by molarity) in DMSO (Final 10% DMSO concentration) for 60 min at 25 °C with shaking. Cross-linking was quenched by adding 1 M Tris, pH8 to a final concentration of 100 mM Tris and incubated for 15 min at 25 °C with shaking. Proteins were reduced by adding TCEP to 10 mM and incubated at 25 °C for 30 min followed by reduction by adding CAA to 55 mM and incubating in the dark at 25 °C for 30 min. Samples were then diluted with 100 mM TEAB, pH 8.5 and digested by adding 1 µg of Lys-C (Wako) and incubated for 4 h at 25 °C with shaking. 1 µg of trypsin (Pierce) was then added and incubated for 16 h at 25 °C with shaking. Trypsin was quenched by acidification to 1% TFA. Digested peptides were desalted using an Oasis 10 mg HLB cartridge (Waters). Cartridges were activated with 200 µL of 100% acetonitrile, then equilibrated twice with 200 µL of 0.1% formic acid in water. Digested peptides were loaded onto the cartridge twice, then washed the cartridge twice with 200 µL of 0.1% formic acid in water and eluted with 200 µL 80% acetonitrile with 0.1% formic acid. Eluted peptides were passed through a C8 stage tip, then vacuum centrifuged to dryness. Dried peptides were stored at −20 °C.

Cross-linked peptides were resuspended in 0.5% acetic acid, 0.06% TFA, 2% acetonitrile in water for analysis on an Easy-nLC 1200 (Thermo) chromatography system coupled to an Orbitrap Fusion Lumos mass spectrometer (Thermo) using a 50 cm × 75 µm inner diameter Easy-Spray reverse phase column (C-18, 2 µm particles, Thermo) over a 60 min gradient from 0.1% formic acid in water to 40% acetonitrile with 0.1% formic acid. MS acquisition was performed with MS1 using Orbitrap 60 K resolution with scan range 350–1650 *m*/*z*. Precursor ions with 3–8 positive charge were selected for MS2 CID fragmentation with a normalized collision energy of 30% and an Orbitrap analyzer at 30 K resolution. MS3 HCD fragmentation was triggered based on targeted mass difference of DSSO (31.9721) for 4 dependent scans with a normalized collision energy of 30% and Ion Trap analyzer in rapid mode.

Thermo raw files were searched against fasta file containing NS2B and NS3 sequence using Metamorpheus v0.0.318 [32] with a calibration search with precursor mass tolerance of 10 ppm and product mass tolerance of 20 ppm. Cross-link search was performed for DSSO on K,S,T,Y amino acids for MS2 CID and MS3 HCD scans with 3 maximum missed cleavages, trypsin protease and fixed modification for carbamidomethyl (C) and variable modifications for oxidation (M), deamidation (N,Q) DSSO hydrolyzed by water and hydrolyzed by Tris (protein N-terminus, K, S, T, Y), DSSO alkene and thiol (protein N-terminus, K, S, T, Y). Cross-links from interlinks and Intralinks result files were filtered for q-value ≤ 0.01. Venn diagrams were made using Venny 2.1.0 [33]. Cross-links and cross-link distances were mapped to and calculated based on crystal structures using Xlink Analyzer v1.1.4 [34] with UCSF Chimera v1.15 (build 42258) [35]. The raw files for the crosslinking MS are deposited on JPOST server [36].

### 4.10. 3D Structure Prediction by AlphaFold

Predicted structures for DENV4 NS2B, NS3 and NS2B-NS3 were computed by AlphaFold2 [37] via ColabFold. [38] For NS2B only and NS3 only predictions, individual protein sequences were run through AlphaFold2 MMseqs2 with a number of models set to 5, homooligomer set to 1. For NS2B-NS3 complex structure prediction, protein sequences were run through AlphaFold2_advanced, with a number of models set as 5, a maximum number of recycles set as 3 with homo-oligomers set as 1:1.

## Figures and Tables

**Table 1:** Data collection and refinement statistics.

Data Collection Statistics	PDB ID: 7VMV
Wavelength (Å)	1.0
Resolution range (Å)	43.330–3.351 (3.471–3.351)
Space group	F 2 3
Unit cell a, b, c α, β, γ, (Å) (^o^)	259.984 259.984 259.984 90 90 90
Total reflections	36,553 (3535)
Unique reflections	20,533 (2084)
Multiplicity	1.8 (1.7)
Completeness (%)	97.65 (99.43)
Mean I/sigma (I)	10.00 (1.36)
Wilson B-factor (Å^2^)	123.70
^a^ R_merge_	0.04594 (0.5728)
R-meas	0.06497 (0.8101)
R-pim	0.04594 (0.5728)
CC1/2	0.998 (0.512)
CC *	1 (0.823)
**Refinement statistics**	
Reflections used in refinement	20,504 (2079)
Reflections used for R-free	988 (121)
^b^ R_work_	0.2258 (0.3606)
^c^ R_free_	0.2628 (0.3576)
Number of non-hydrogen atoms	6046
macromolecules	6046
Protein residues	852
^d^ RMSD (bonds) (Å)	0.004
RMSD (angles) (^o^)	0.98
Ramachandran favored (%)	92.70
Ramachandran allowed (%)	7.18
Ramachandran outliers (%)	0.12
Rotamer outliers (%)	3.63
Clashscore	9.22
Average B-factor	138.53
macromolecules	138.53
Number of TLS groups	8

Statistics for the highest-resolution shell are shown in parentheses. ^a^ R_merge_ = ∑|I_j_ − < I > |/∑I_j_, where I_j_ is the intensity of an individual reflection, and < I > is the average intensity of that reflection. ^b^ R_work_ = ∑||F_obs_| − |F_calc_||/∑|F_obs_|, where F_obs_ denotes the observed structure factor amplitude, and F_c_ the structure factor amplitude calculated from the model. ^c^ R_free_ is as for R_work_ but calculated with 5% of randomly chosen reflections omitted from the refinement. ^d^ RMSD, root mean square deviations.

**Table 2:** Primers used in the experiment.

Target	Purpose	Orientation	Sequence
eNS2B_47_NS3Pro	S135A Mutation	Forward	ACCCGGAACGGCGGGCTCTCCTA
		Reverse	TTGAAATCCAGAGTTACTGCTCCAATTTCTC
eNS2B_47_NS3Pro	Linearisation	Forward	TCAGGAGCTCTGTGGGACG
		Reverse	TCTTTGTGTTTTCACTTGTGC
eNS2B_47_NS3Pro	Fragment Insertion	Forward	GTGAAAACACAAAGATAAGAATTCTTGTACACGGCCGCATAATCGAAATTAATACGACTCACTATAGGGGAATTGTGAGCGGATAACAATTCCCCATCTTAGTATATTAGTTAAGTATAAGAAGGAGATATACATATGTCAGGAGCTCTGTGG
		Reverse	CCACAGAGCTCCTGACATATGTATATCTCCTTCTTATACTTAACTAATATACTAAGATGGGGAATTGTTATCCGCTCACAATTCCCCTATAGTGAGTCGTATTAATTTCGATTATGCGGCCGTGTACAAGAATTCTTATCTTTGTGTTTTCAC

## Data Availability

The crosslinking mass spectrometry data has been deposited with JPOST number with JPOST ID: JPST001547 and PXID: PXD033312. The crystallographic data has been deposited to protein data bank with the dataset ID: D_1300024934 and PDB id: 7VMV.

## Supplementary Materials

The following supporting information can be downloaded at: https://www.mdpi.com/article/10.3390/v14071440/s1.
