# Supplementary material for: Dynamic Interactions of Post Cleaved NS2B Cofactor and NS3 Protease Identified by Integrative Structural Approaches
Source: Viruses. 2022 Jun 30;14(7):1440. doi: 10.3390/v14071440 (PMC9323329; doi:10.3390/v14071440)
Supplement: Supplementary file 1 [file viruses-14-01440-s001.zip › viruses-1714512-supplementary.pdf]

Supplementary Figures

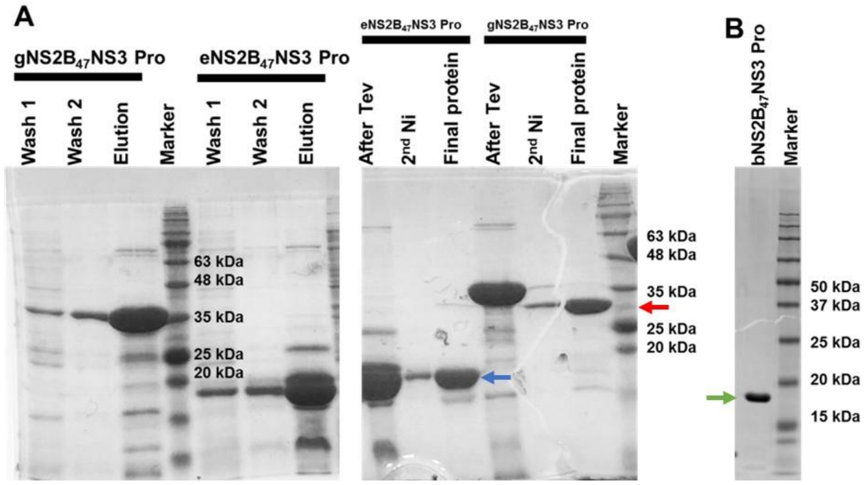

Supplementary Figure S1. SDS PAGE analysis of purified g-, e-, and b-NS2B$_{47}$NS3Pro proteins. The g-NS2B$_{47}$NS3Pro proteins, NS3Pro of e-NS2B$_{47}$NS3Pro proteins and NS3 of b-NS2B$_{47}$NS3Pro proteins have expected molecular weights of 25.7 kDa, 19.7 kDa and 19.4kDa respectively. The g-NS2B$_{47}$NS3Pro proteins, NS3Pro of e-NS2B$_{47}$NS3Pro proteins and NS3 of b-NS2B$_{47}$NS3Pro are indicated by the red, blue and green arrows respectively.

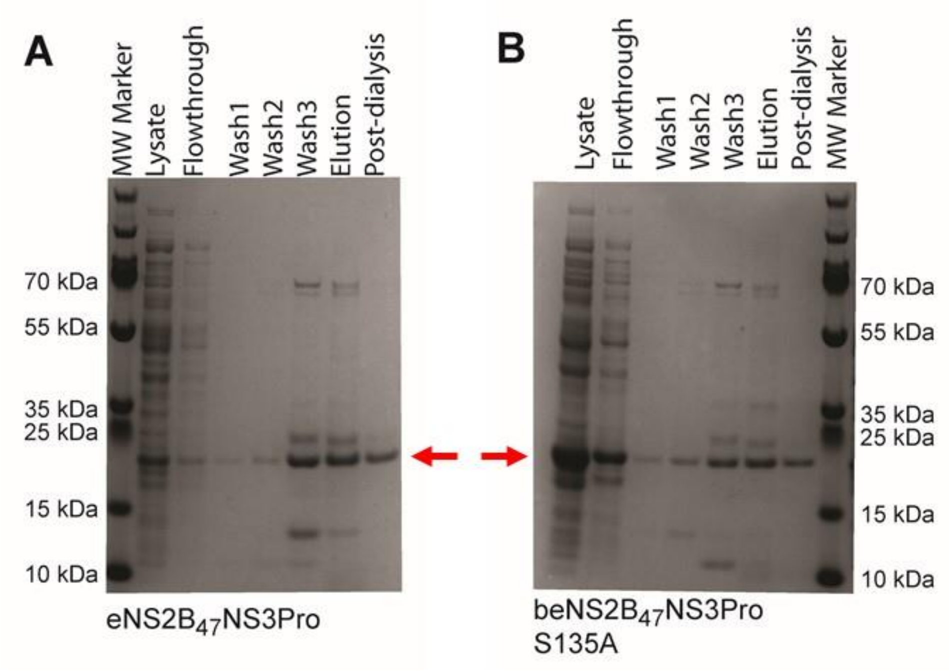

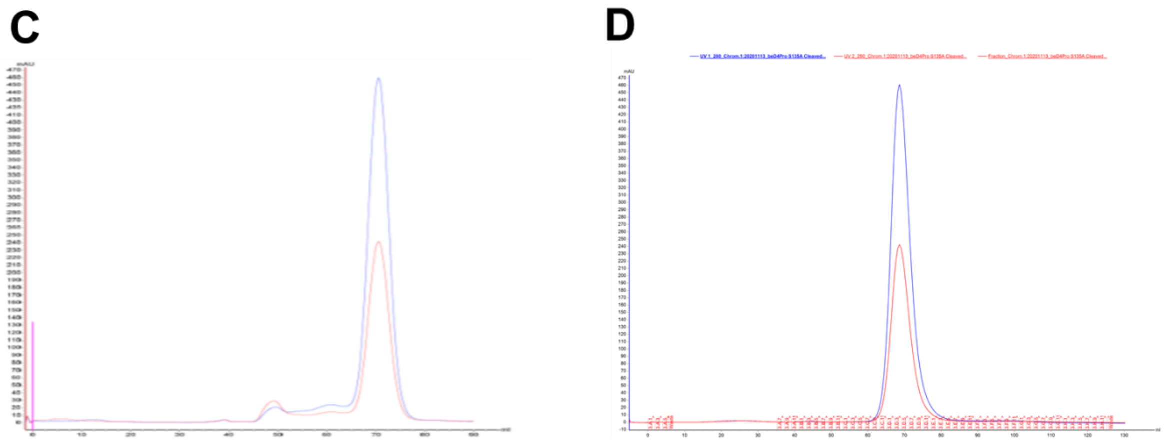

Supplementary Figure S2. SDS PAGE analysis of (A) eNS2B47NS3Pro and (B) beNS2B47NS3Pro S135A. The expected molecular weight of the NS3Pro is 19.7kDa. The bands of interest are indicated by the arrows. Gel filtration profiles of (C) eNS2B47NS3Pro and (D) beNS2B47NS3Pro S135A.

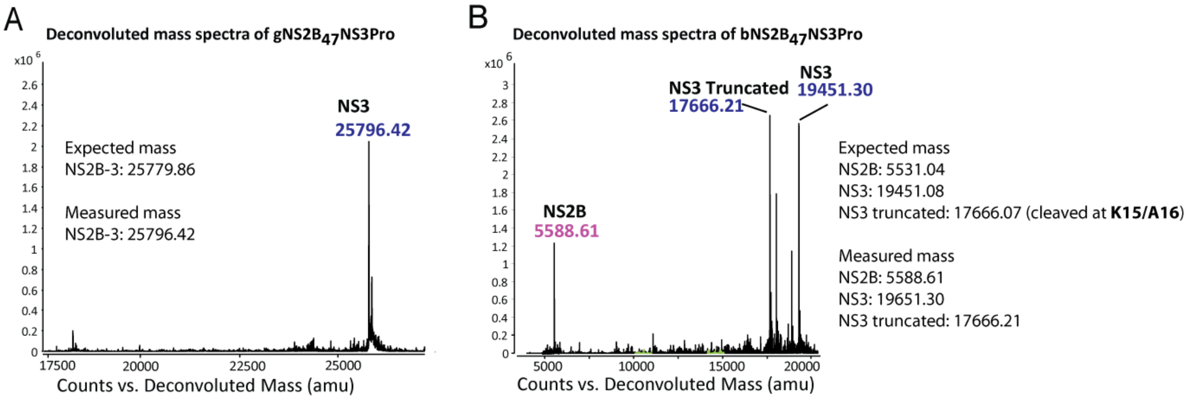

Supplementary Figure S3. Molecular weight determination of DENV4 recombinant NS2B-NS3 protease. (A) Deconvoluted mass spectra of gNS2B47NS3Pro. No internal cleavage of observed. (C) Deconvoluted mass spectra of bNS2B47NS3Pro. No truncated NS2B was observed. A portion of NS3 protease was truncated at N-terminus.

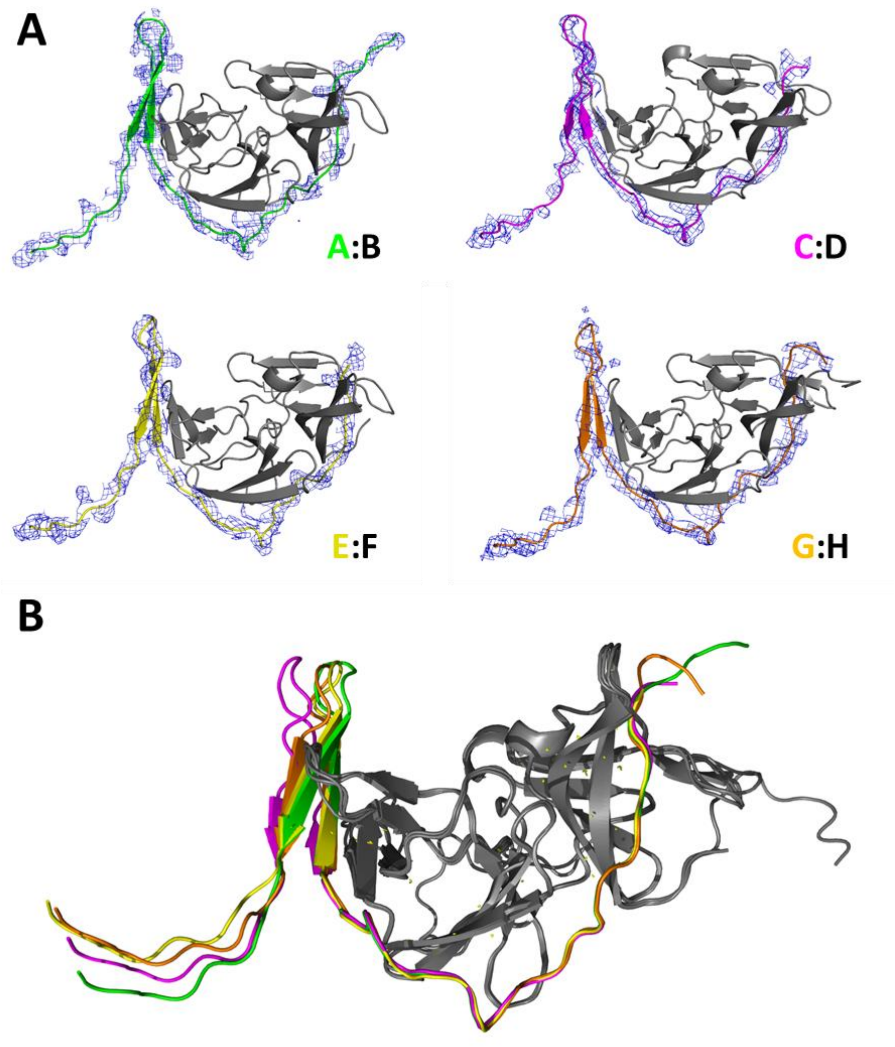

Supplementary Figure S4. (A) Electron density map of NS2B co-factor for the four eNS2B$_{47}$NS3Pro molecules. The NS3 domains are in grey and NS2B in green, magenta, yellow and orange. The electron density map (2Fo-Fc) are coloured in blue and contoured at 1σ. (B) Superimposition of four molecules of eNS2B$_{47}$NS3Pro.

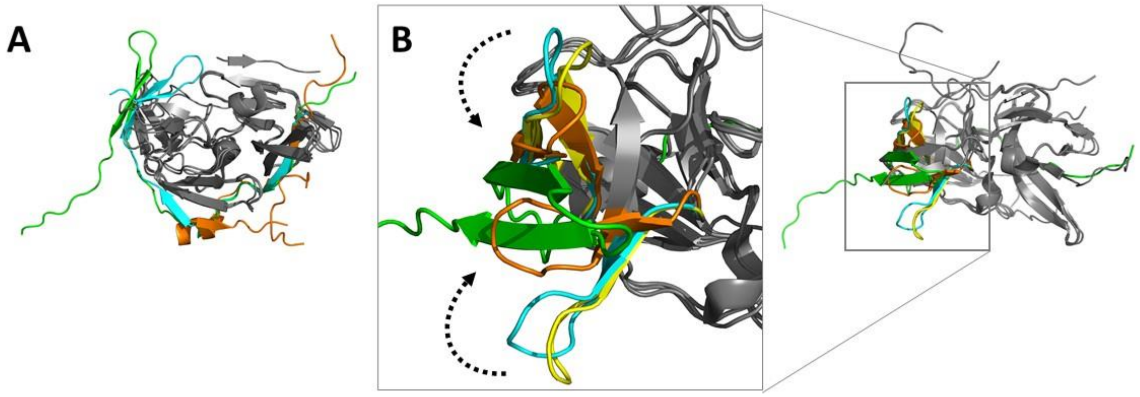

Supplementary Figure S5. Comparison of eNS2B$_{47}$NS3Pro with published structure of Flavivirus NS2B-NS3 protease. (A) Conformation of NS2B cofactor in various conformation. NS2B cofactor of eNS2B$_{47}$NS3Pro in partially closed conformation in green, NS2B cofactor of DENV2 gNS2B$_{47}$NS3 Pro (PDB:2FOM) in open conformation in orange and NS2B cofactor of DENV3 gNS2B$_{47}$NS3 Pro (PDB:3U1I) in closed conformation in cyan. (B) Two loops (residues 111-126 and residues 151-162) in the NS3 act as hinges to mediate the conformation changes of NS2B-NS3 protease. The NS2B cofactor of eNS2B$_{47}$NS3Pro are shown in green. While the loops are shown in yellow for eNS2B$_{47}$NS3Pro, orange for DENV2 gNS2B$_{47}$NS3 Pro (PDB:2FOM) in open conformation, cyan for DENV3 gNS2B$_{47}$NS3 Pro (PDB:3U1I) in closed conformation.

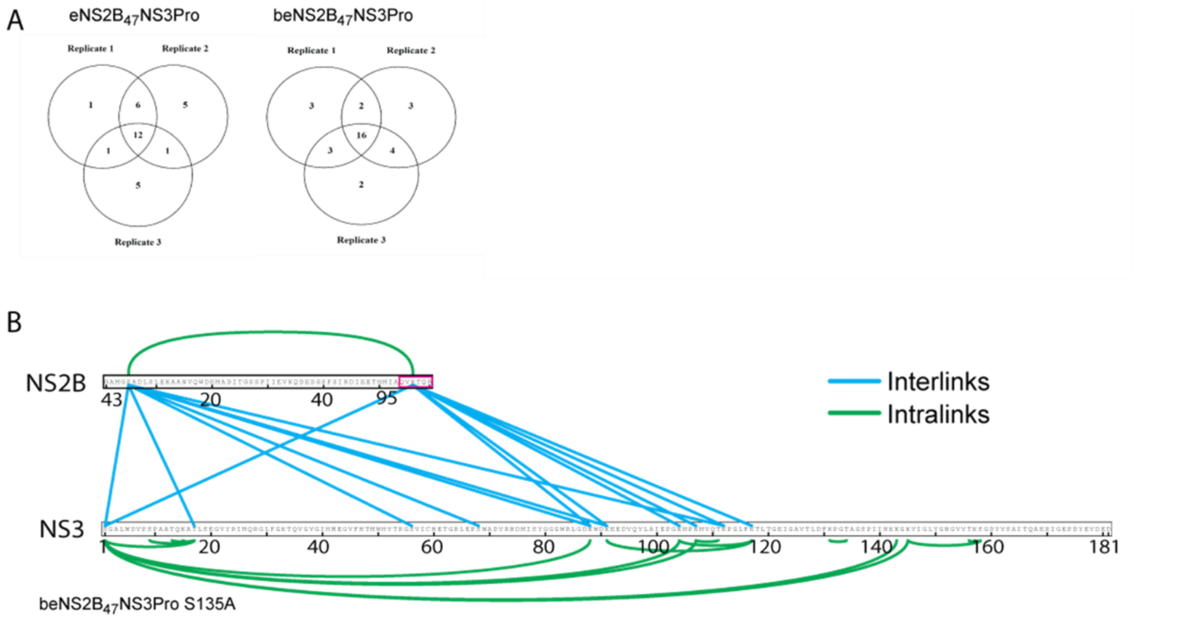

Supplementary Figure S6. Mapping of dynamic interactions between NS2B and NS3 via crosslinking mass spectrometry. (A) Venn diagram showing overlap of cross-links identified between three replicates. (B) Residues from NS2B and NS3 that are mapped as intra and inter molecular crosslinks in beNS2B$_{47}$NS3Pro S135A.
